# Identification of Functional Regulatory Residues of the ****β****-Lactam Inducible Penicillin Binding Protein in Methicillin-Resistant *Staphylococcus aureus*

**DOI:** 10.1155/2013/614670

**Published:** 2013-07-29

**Authors:** Andreas N. Mbah, Raphael D. Isokpehi

**Affiliations:** Center for Bioinformatics & Computational Biology, Department of Biology, Jackson State University, Jackson, MS 39217, USA

## Abstract

Resistance to methicillin by *Staphylococcus aureus* is a persistent clinical problem worldwide. A mechanism for resistance has been proposed in which methicillin resistant *Staphylococcus aureus* (MRSA) isolates acquired a new protein called **β**-lactam inducible penicillin binding protein (PBP-2′). The PBP-2′ functions by substituting other penicillin binding proteins which have been inhibited by **β**-lactam antibiotics. Presently, there is no structural and regulatory information on PBP-2′ protein. We conducted a complete structural and functional regulatory analysis of PBP-2′ protein. Our analysis revealed that the PBP-2′ is very stable with more hydrophilic amino acids expressing antigenic sites. PBP-2′ has three striking regulatory points constituted by first penicillin binding site at Ser25, second penicillin binding site at Ser405, and finally a single metallic ligand binding site at Glu657 which binds to Zn^2+^ ions. This report highlights structural features of PBP-2′ that can serve as targets for developing new chemotherapeutic agents and conducting site direct mutagenesis experiments.

## 1. Introduction

Methicillin resistance (MR) by *Staphylococcus aureus *is a persistent clinical problem affecting many geographic locations worldwide [1–4]. Glycopeptides such as vancomycin and teicoplanin are often the choice in treating infections associated with methicillin-resistant *S. aureus *(MRSA), at times with little success [5]. The resistance of methicillin by *Staphylococcus aureus* has been documented to depend on several factors such as temperature [6], pH [7], NaCl concentration, and inoculum size [6, 8]. Even though methicillin-resistant staphylococci produce penicillinase, blocking of this enzyme do not affect the level of methicillin resistance [9]. The history of ever-increasing resistance among MRSA strains suggests that they are likely to be more prevalent in the future, thus severely restricting treatment options [10].

Penicillin-binding proteins (PBPs) are enzymes commonly expressed by MRSA during peptidoglycan synthesis, cell growth, and morphogenesis. The PBPs are inhibited by *β*-Lactam antibiotics such as methicillin and vancomycin by interrupting the biochemical functions at the D-Ala-D-Ala terminus of the peptidoglycan precursor [5, 11]. The expressions of PBPs in MRSA have been well documented in previous studies, with PBP-2 and PBP-3 proposed as the lethal targets for *β*-lactams action [12–14]. However, another mechanism for methicillin resistance have been reported in which MRSA isolates have acquired a new PBP protein called *β*-lactam inducible penicillin binding protein (PBP2a or PBP-2′) [1]. The induction of PBP-2′ occurs only in the presence of penicillinase plasmid, and PBP-2′ can be produced constitutively in MRSA which had lost the penicillinase plasmid [1, 15]. The PBP-2′ protein has low affinity for penicillin and most other *β*-lactam antibiotics. The PBP-2′ is capable of substituting for other PBPs during cell wall synthesis after *β*-lactams antibiotics have inhibited them [16]. In this model, PBP-2′ acts as a surrogate enzyme capable of taking over the normal functions of staphylococcal PBPs in cell wall biosynthesis. The ability of PBP-2′ to affect cell wall synthesis in the presence of methicillin requires cooperation from the transglycosylase domain of the native PBP-2 [17, 18]. 

 The amino acid sequence of PBP-2′ is similar to those of the shape-determining protein (PBP-2) and septum-forming (PBP3) of *Escherichia coli* [10]. This support the idea that PBP-2′ might have evolved as a combination of two genes of inducible type I penicillinase gene and a PBP gene which coordinate the expression of beta-lactam-inducible MRSA PBP [19]. The PBP-2′ protein is encoded by the *mecA *gene carried on a large mobile genetic element also known as SCC*mec *[20–22], which integrates it into the chromosome of MRSA strains. However, MRSA PBP-2′ expression is modulated by a transacting factor in response to the presence of the cell wall-active antibiotics such as methicillin, vancomycin, and oxacillin [10]. Thus, PBP-2′ is proposed as a vital contributor in the increased prevalence of MSRA [1, 17, 18].

Presently few therapeutic alternatives exist for the treatment of MRSA infections. This research report presents structural features that regulate the functioning of PBP-2′. These features could be exploited in developing new chemotherapies against MRSA. Our analysis reveals that the PBP-2′ protein has three striking regulatory points constituted by first penicillin binding site at Ser25, second penicillin binding site at Ser405 and finally a single metallic ligand binding site at Glu657 which binds to Zn^2+^ ions. These structural features can be exploited as novel targets for developing new drugs against MSRA.

## 2. Materials and Method

### 2.1. Sequence Retrieval, Amino Acid, and Physicochemical Parameters Analysis

The beta-lactam-inducible penicillin binding protein (PBP-2′) (UniProt ID: P07944 *|* PBP_STAAU) reviewed sequence was retrieved from UniProt protein database (UniProt release 2011_11) (http://www.uniprot.org/). The amino acid composition of the sequence was computed using the ProtParam tool (http://www.expasy.ch/cgi-bin/protparam). The ProtParam tool was also used to compute the physicochemical parameters such as theoretical isoelectric point (Ip), molecular weight, total number of positive and negative residues, extinction coefficient, half-life, instability index, aliphatic index, and grand average hydropathy (GRAVY). The percentages of hydrophobic and hydrophilic residues were calculated from the primary structure analysis and the hydrophobicity plot was done using both Hopp-Woods and Kyte-Doolottle scale for possible antigenicity. 

### 2.2. Conserved Domain Search, Homology Modeling, and Visualization of 3D Structure

The possible conserved domains regulating the functional mechanism of PBP-2′ protein were analyzed using the NCBI public server at http://www.ncbi.nlm.nih.gov/Structure/cdd/wrpsb.cgi, UniProt protein database (UniProt release 2011_11) (http://www.uniprot.org/), and Pfam database at http://pfam.sanger.ac.uk/search/. Their functional units and domain residues were identified and documented. The three-dimensional (3D) structure of (PBP-2′) encoded protein was modeled using the PDB template 1mwu (Chain A). The three-dimensional (3D) structure of the gene was determined using the following servers; SwissModel server (http://www.swissmodel.expasy.org/) and the Phyre/Phyre2 server (http://www.sbg.bio.ic.ac.uk/phyre2/html/page.cgi?id=news) [23]. The quality of the model was evaluated with Ramachandran plot data, based on the phi-psi torsion angles of all the residues in the model using DeepView-Swiss-PdbViewer (http://spdbv.vital-it.ch/). The Ramachandran plot obtained from DeepView was further assessed using Ramachandran plot 2 assessment server (http://dicsoft1.physics.iisc.ernet.in/rp/). The Rasmol tool (http://www.openrasmol.org/) was used in visualizing the modeled 3D structures and the distribution of the secondary structures. The three-dimensional (3D) LigandSite residues and the predicted ligand of the PBP-2′ protein were determined using 3DLigandSite server at (http://www.sbg.bio.ic.ac.uk/3dligandsite/) [24]. The Rasmol tool was further employed in locating the positions of the ligand binding sites and the ligand on the 3D structure with particular attention to the regulatory points for PBP-2′. 

## 3. Results and Discussion

### 3.1. Amino Acid Content and Physicochemical Parameters

The analysis suggests that this protein is hydrophilic due to the presence of high polar amino acid residues (53.3%) against nonpolar (hydrophobic) amino acids residues (35.1%) (Table 1). The protein can be described as moderately hydrophilic. The hydrophobic residues are usually found in the core of most proteins, and they help in stabilizing the proteins through the numerous *van der Waal* interactions [25]. The hydrophilic residues are located mostly at the surface active sites of proteins, where they interact with other polar residues or with water molecule. The PBP-2′ protein is made of 670 amino acid residues with an average molecular weight of 76463.2 Da. The analysis indicates that there are more Lys, Ile, Asn, Asp, Gly, and Ser in that order (Table 1). The atomic composition (10812 atoms) consists of 3415 carbons (C), 5428 hydrogen (H), 912 nitrogen (N), 1039 oxygen (O), and 18 sulfur atoms with a molecular formula of C_3415_H_5428_N_912_O_1039_S_18_ (Table 2). The 18 sulfur atoms were constituted by methionine residues present in the primary structure. The computed pI (9.09) [pI > 7] indicates that the protein is basic in nature. The isoelectric point (pI) indicates the pH at which the protein surface is covered with charge [26], and the net charge of this protein is positive. The high number of positively charged residues (Arg + Lys = 105) against the total number of negatively charged residues (Asp + Glu = 90) is the main contributing factor to the positive charge. At a given pI, proteins are stable and compact; thus, this parameter will be useful for developing buffer systems for purification of this protein by isoelectric focusing techniques [27]. 

The ProtParam extinction coefficient at a wavelength of 280 nm measured in water is favorable because proteins are able to absorb strongly at this wavelength than other substances that may be commonly found in the solution. The extinction coefficient for this protein was computed with respect to Trp and Tyr present in the primary structure. The extinction coefficient of 93630 M^−1^ cm^−1^ at 280 nm wavelength computed for this protein was constituted by individual contributions from Trp (1.0%) and Tyr (5.5%), respectively. This suggests that PBP-2′ protein can be analyzed using UV spectrum assay [28]. The computed protein concentration and the extinction coefficient will contribute immensely in the quantitative analysis of the protein-protein and protein-ligand interaction of this protein in solutions [28, 29]. The estimated half-life of this protein with Met as the N-terminal of the sequence was 30 (>20) hours. The concentrations of Ala (3.9%), Leu (6.9%), and Val (5.5%) may be contributing to the stability of the protein [30, 31]. The half-life of these 3 mentioned residues had been well documented in the mammalian with values of Ala (4.4 hours), Leu (5.5 hours), and Val (100 hour). In other organisms, these residues are also contributing to the protein stability. In yeast, the half-life was the same (>20 hours) for Ala and Val, and both were the same in *E coli* with values >10 [32].

Based on the predicted instability index, the ProtParam tool indicates that the protein is stable with a value of 30.08. This parameter was computed based on the impact of dipeptides in the protein sequence [33]. This protein is shown to have high aliphatic index, half-life, and large amount of hydrogen atoms (5428) to form strong hydrogen bonds. Such hydrogen bonds are known to impact significant stability to proteins, making them resistance to degradation [26]. Therefore the formation of hydrogen bonds is contributing to its stability. The stability value is a measure of the protein stability in a test tube [26]. A protein of instability index <40 is considered as stable, while those with values >40 are unstable [33].

**Figure 1:**
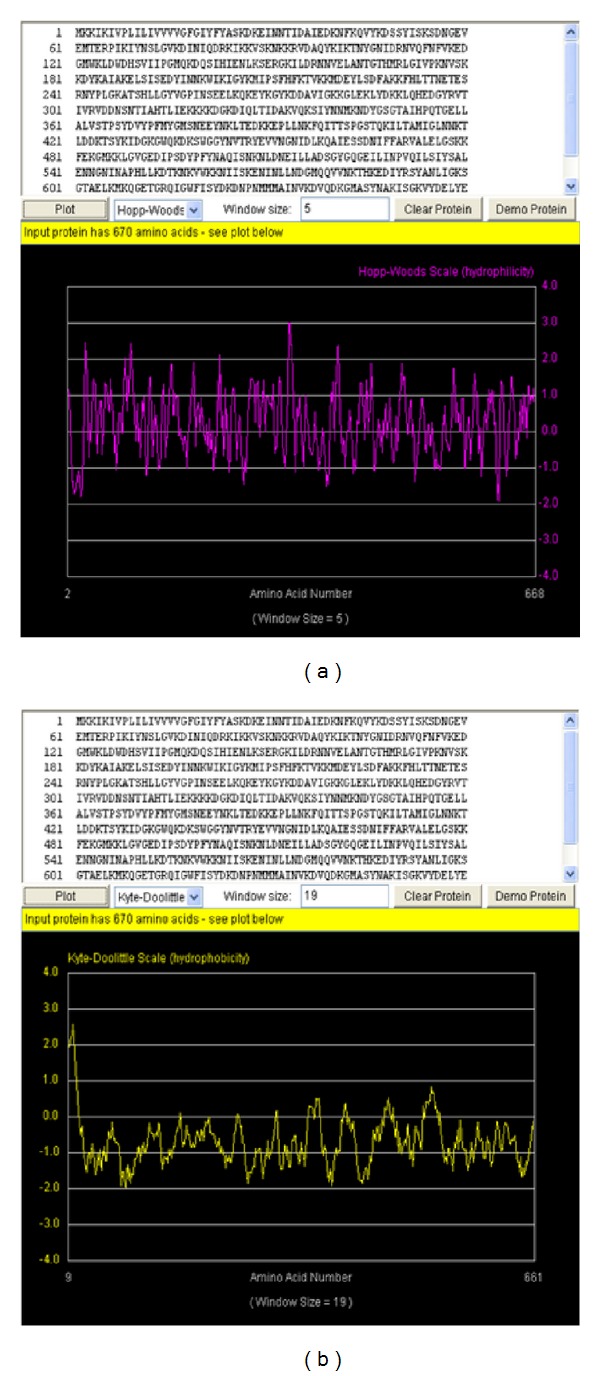
The hydropathy plot for PBP-2′ protein. The yellow plot (b) is the Kyte-Doolittle hydrophobicity plot. Sections of the plot with high values >0.0 are highly hydrophobic or membrane spanning segments. The magenta plot (a) is the Hopp-Wood hydrophilicity plot. Higher values above >0.0 predict rich charge exposed regions with potential antigenic site. PBP2′ gene shows potential antigenic sites with values ≥2. Above the plots are the PBP2′ amino acids sequence with 670 residues.

The aliphatic index (AI) of a protein is the relative volume occupied by the aliphatic side chains (alanine, valine, isoleucine and leucine) and is taken as contributor to the increase thermal stability of globular proteins. The aliphatic index computed for this protein was 82.76 using a formulated rule illustrated by [34]. This high aliphatic index indicates that the protein can be stable within a wide range of temperature. Proteins with low thermal stability turn to be more structurally flexible. The grand average of hydropathy (GRAVY) is the computed sum of hydropathy values of all the amino acids, divided by the number of residues in the sequence [35]. The very low GRAVY index (−0.698) of this protein indicates that the protein is very reactive in water, which might be a contributing factor to MRSA virulence and pathogenicity. The Hopp-Woods scale identified three regions on this polypeptide predicted to be highly hydrophilic. These hydrophilic regions are exposed on the surface and may possibly be antigenic [36]. They are shown to have peak values greater than 0 (Figure 1). This indicates that this protein can be served as a possible vaccine target.

### 3.2. Conserved Domain and Functional Regulatory Point Analysis of MRSA PBP-2′ Gene

**Figure 2:**
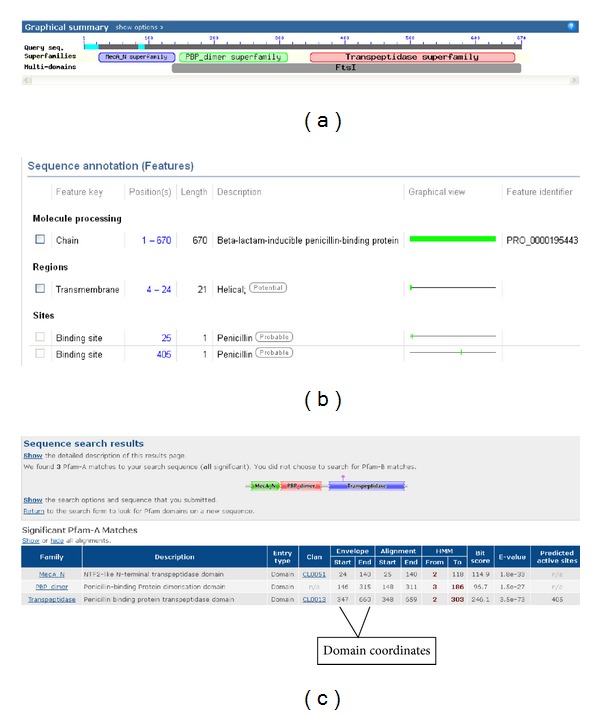
Visualization of the functional domains of PBP-2′ protein using NCBI, UniProt, and Pfam domain tools. The position and span of each domain unit across the protein are shown. The span of residues contributing to the function of each domain is shown including the regulatory points. (a) is the functional domain from NCBI while (b) is the Uniprot database annotation. (c) shows the domain verification with Pfam annotation.

Methicillin-resistant *Staphylococcus aureus* is a leading cause of hospital carried infections worldwide and is a leading community-associated pathogen [37]. These *Staphylococcus* strains are cross-resistant to virtually all *β*-lactam antibiotics clinically used worldwide [38]. The MRSA clinical strains are often multidrug resistant, thus limiting the therapeutic options for staphylococcal infections [39]. The conserved domains in proteins are responsible for many important biological functions within the cellular process of an organism [40–43]. The conserved domain search for PBP-2′ protein revealed 3 functional distinct domains within its amino acids sequence. These domains are MecA_N (residue 24–140), PBP-dimer (residue 146–315), and Transpeptidase (residue 347–660) (Figure 2).

**Figure 3:**
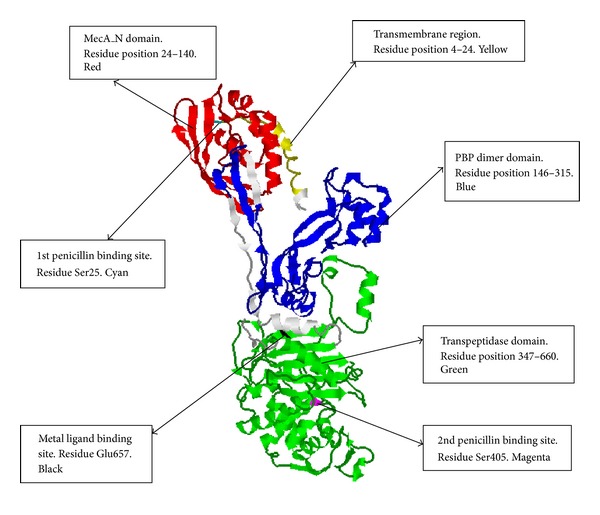
The functional domains distribution and position of regulatory amino acid residues on PBP-2′ protein 3D structure.

**Figure 4:**
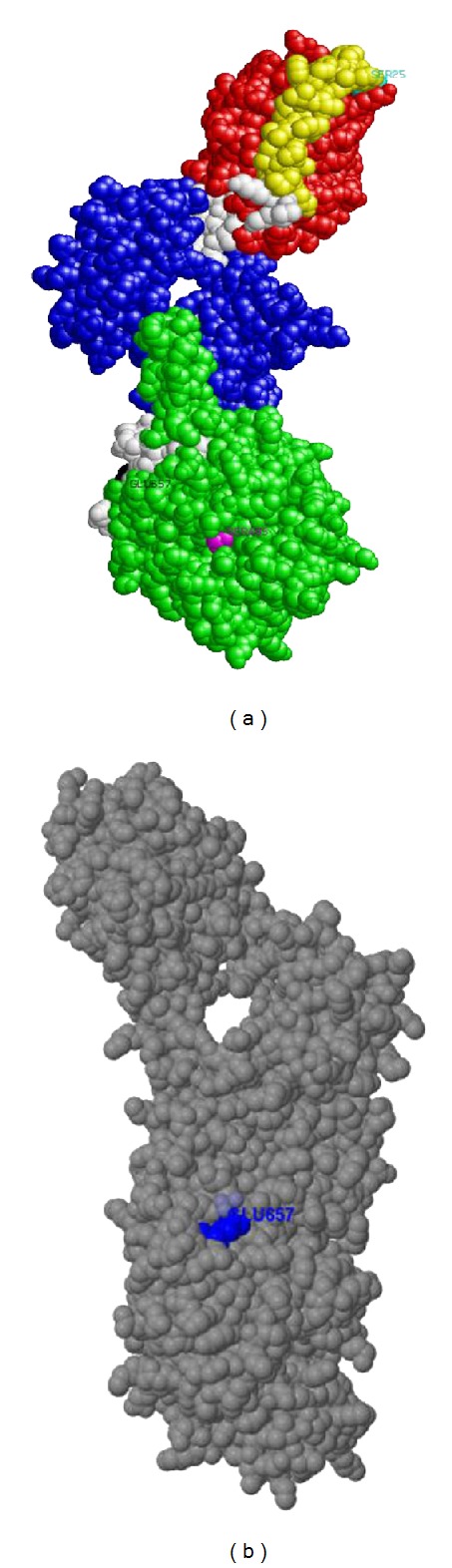
The position of the regulatory points and the binding of Glu657 residue to Zn^2+^ ion on PBP-2′ protein folded structure. (a) On the folded structure the first penicillin binding site Ser25 (color cyan) and the metallic ligand binding site Glu657 (color black) are located on the surface while the second penicillin binding site Ser405 (color magenta) is not exposed on the surface but situated in an active site cavity. (b) This shows the Zn^2+^ ion (color grey) binding to the Glu657 residue (color blue) on the protein surface.

The crystal structure of PBP-2′ gene has not yet been determined here we present the 3D modeled structure of the protein using 1 mwu (Chain A) as template with 94% of the residues modeled at >90% confidence level. The Rasmol tool was used for visualizing the tertiary structure of the PBP-2′ protein. The secondary structure components composed of 31 helices, 41 sheets, 60 turns, and 5478 hydrogen bonds shown with dotted line. The MecA_N domain spans residue position 24–140, and it is depicted red in color (Figures 3 and 4) and responsible for expressing the *mecA* gene. The phenotypical characteristics of MRSA are due mainly to the presence of *mecA*, which encodes a penicillin-binding protein (PBP-2′) with reduced affinity for *β*-lactams [13, 19]. The genetic determinant of PBP-2′, the *mecA* gene, is not native to *S. aureus* but was reported to be imported from an unidentified extraspecies source [44]. The *mecA* gene is localized in a large heterologous chromosomal cassette, the SCC*mec* element [45], and some MRSA strains carry upstream to the *mec*A gene the regulatory genes *mec*I*-mec*R1 encoding for a repressor and a sensor/inducer of the *mec*A expression [46]. Because *mec*I-*mec*R1 induction of *mec*A expression is not fast enough, functional *mec*I and *mec*R1 genes render the cell phenotypically susceptible in the presence of *mec*A [46, 47]. In vitro, the deletion of the *mec*I gene increases the resistance levels to *β*-lactams in staphylococci [46–48]. This suggests that complete resistance to *β*-lactams by MRSA might be contributed by nonfunctional *mec*I*-mecR*1 regulatory system [46, 47]. There are documented evidences of the accumulation of point mutations in the *mec*I coding sequence in several MRSA strains [49–53]. 

However, there is no clear relationship between the amounts of *mec*A transcript or PBP2′ protein and the phenotypic level of resistance [54, 55]. MRSA strains are also classified based on the type of SCC*mec* element they carry because the same lineage may be associated with several SCC*mec* types [56, 57]. Also MRSA strains are known to show strain-to-strain variation in resistance level, genetic backgrounds and in their SCCmec structures that carries the resistance mecA gene [58]. In *S. aureus,* three major *mec* classes have been described based on the presence of insertion sequences and intact or disrupted *mec*I*-mec*R1 sequences. The class A has intact sequences for *mec*I*-mec*R1 whereas classes B and C have no *mec*I and have partially deleted *mec*R1 due to the integration of insertion sequences in the regulatory region of *mec*A gene. Based on the SCC*mec* types, the *mec* gene complexes are grouped into three classes as follows: class A (SCC*mec* types II, III, and VIII); class B (SCC*mec *types I, IV, and VI), and class C (SCC*mec* types V, and VII) [39]. Similarly the epidemicity of MRSA strains does not properly correlate with the *mec*I*-mec*R1 functionality as well. This has been reported with the nosocomial MRSA clones from the ST5-II and the ST239-III which were characterized to SCC*mec* types II and III, respectively, even though they have a complete *mec*I-*mec*R1 locus [56].

The *mec*A domain also contains one of the penicillin binding sites located on amino acid residue Ser25 (color cyan) which is considered as one of the active sites of PBP-2′ (Figures 3 and 4). It is located on the loop of the secondary structure and on the surface of the PBP-2′ folded structure. This suggests that this site might be very unstable for penicillin binding [59, 60]. Boronic acid bounds and inhibits the nucleophilic serine of the active site and thus mimicking the transition state of the enzymatic reaction involved in the catalytic mechanism of penicillin-binding proteins [61]. The Ser25 is one of the key residues involved in the catalytic mechanism of penicillin-binding proteins, suggesting that the PBP-2′ function might be regulated at this amino acid residue using amino acid substitutions, combined with site-directed mutagenesis studies [62].

The second domain is the PBP dimer which is constituted by amino acid residues 146–315 and is represented in blue (Figures 3 and 4). There is evidence suggesting that PBPs exist in dimeric forms [63]. The PBPs anchor to cytoplasmic membranes by an amino-terminal transmembrane segment [64, 65], and the dimer formed is less soluble than monomeric PBPs [63]. This suggests that the PBP-2′ dimeric form is more tightly associated with the peptidoglycan layer and/or the outer membrane than the monomeric form. The dimeric PBPs are suggested to be located both in the outer and inner membrane fractions [66]. This indicates that the fraction of PBP-2′ not associated with the cytoplasmic membrane could be the active enzyme. The dimeric form of PBP is not linked by disulfide cross-links [67]. This is in agreement with our analysis on the amino acid content of PBP-2′ which lacks cysteine residues in its amino acid sequence length (Table 1). Nevertheless, these dimers involve strong noncovalent interactions [67]. There is no clear documentation on the physiological role regarding dimerization of PBP, and it remains speculative. Irrespective of this, there is suggestion that the existence of the dimer is a well monitored and carefully regulated event and recent models of peptidoglycan synthesis imply that PBP acts as a dimer within a multienzymatic complex [68, 69].

The third domain is the transpeptidase domain constituted by amino acid residue 347–660 (color green) (Figures 3 and 4). Several lines of evidence indicate that transpeptidase activity is the essential function of PBP2 compensated by PBP-2′. This supports the genetic evidence that PBP-2′ has transpeptidase activity (TPase activity) [70]. Formerly the TPase activity of PBP-2′ was assumed based on homology with other PBPs and models of methicillin resistance in *S. aureus* [19]. The PBP-2′ is currently classified as class B PBP with a TPase domain and a penicillin-insensitive second domain whose function is not known [71]. It is generally supported that membrane-bound transpeptidase which catalyzes the cross-linking of neighboring amino acid side chains during cell wall peptidoglycan synthesis is a major target for *β* lactam action and that inhibition of transpeptidase enzyme leads to cell death [72, 73]. Further, the transpeptidase domain is required for cell wall protein anchoring and virulence in MRSA [74]. The transpeptidase domain also contains two regulatory point for the PBP-2′ gene. This includes the second penicillin binding site located at residue Ser405 (color magenta). The second penicillin binding site is also known as the active site or catalytic residue of the enzyme (Figures 3 and 4) [75]. 

As indicated above for Ser25 (first penicillin binding site), Ser405 might be regulated using amino acid substitutions combined with site-directed mutagenesis studies [62]. The Ser405 active site is very stable in the active fold linking with other residues through a network of hydrogen bonds. There is only one metallic ligand binding residue at Glu657 depicted black in color (Figures 3 and 4). It is exposed on the surface of the protein for easy binding to free metallic ligand such as Zn^2+^ ions (Figures 3 and 4).The Glu residue has been document as one of the essential residue sites involved when metals bind to proteins [76, 77], and Ser is also known as one of the key phosphorylation residues in proteins [78–80]. Metallic ions have been demonstrated as essential contributors to protein phorphorylation [81–85]. This suggests that in PBP-2′ gene transpeptidase activity might involved or could be regulated by phorphorylation through the binding of metallic ions as ligands. Our analysis indicates that Zn^2+^ ions might be the key metallic ligand regulating the transpeptidase activity in MRSA. Polyoxotungstates in combination with beta lactam antibiotics make the MRSA strains more susceptible [86]. Also polyoxotungstates can be interchangeable substituted by transitional metal [87]. Therefore, the transitional metallic Zn^2+^ ions could be regulated by using appropriate ligands to improve the susceptibility of the MRSA to common *β* lactam antibiotics. This finding is an important contribution in identifying new chemotherapeutic strategies for MRSA.

## 4. Conclusions

Previous studies and computer aided design drug screenings have been focusing mostly on penicillin binding protein 2A (PBP2A) as the virulent factor of most of the MRSA associated infections [38]. Another key component of methicillin-resistant mechanism is an acquired penicillin-binding protein (PBP), PBP-2′, which has unusually low affinity for all *β*-lactam antibiotics and most other antibiotics [88]. We have elucidated the distinctive structural features of PBP-2′ protein which can be exploited as chemotherapeutic targets for MSRA. The PBP-2′ consist of 3 domains of MecA, PBP dimer and transpeptidase. The structural regulatory points consist of penicillin binding sites at Ser25, Ser405, and a metallic ligand binding site at Glu657. These findings offer new insight into the dynamic and functional determinants of PBP-2′ in developing novel chemotherapeutic agents for MSRA. 

## Figures and Tables

**Table 1:** Amino acid composition of PBP-2′ computed using ProtParam server.

Amino acid*	Composition (%)	Hydrophilic (%)	Hydrophobic (%)
Ala	3.9		3.9
Arg	2.1	2.1	
Asn	8.5	8.5	
Asp	7.5	7.5	
Cys	0.0		
Gln	3.4	3.4	
Glu	6.0	6.0	
Gly	7.0		7.0
His	1.6	1.6	
Ile	9.3		9.3
Leu	6.9		6.9
Lys	13.6	13.6	
Met	2.7		
Phe	2.4		
Pro	2.5		2.5
Ser	6.3	6.3	
Thr	4.3	4.3	
Trp	1.0		
Tyr	5.5		
Val	5.5		5.5

Total	100.0	53.3	35.1

*The composition of each amino acid residue is indicated in percentage. The composition of hydrophilic amino acids is 53.3% while hydrophobic amino acids constitute 35.1%. The protein can be described as moderately hydrophilic.

**Table 2:** Physicochemical properties of PBP-2′ computed using ProtParam server.

ProtParam parameters*	Values
No. of amino acids	670
Molecular weight	76463.2 Da
Theoretical pI	9.09
No. of negative charge residues	90
No. of positive charge residues	105
Formula	C_3415_H_5428_N_912_O_1039_S_18_
Extinction coefficient	93630 M^−1^ cm^−1^
Estimated half-life	30 hours
Instability index	30.08
Aliphatic index	82.76
Grand average of hydropathicity (GRAVY)	−0.698
Total number of atoms	10812

*The physicochemical parameters define the protein chemical and physical properties in its native state. The protein has a net positive charge and is basic in nature (pI > 7).
